# Acute neutrophilic vasculitis (leukocytoclasia) in 36 COVID-19 autopsy brains

**DOI:** 10.1186/s13000-024-01445-w

**Published:** 2024-02-15

**Authors:** Roy H. Rhodes, Gordon L. Love, Fernanda Da Silva Lameira, Maryam Sadough Shahmirzadi, Sharon E. Fox, Richard S. Vander Heide

**Affiliations:** 1 Department of Pathology, Louisiana State University Health Sciences Center, 7th Floor, 2021 Perdido Street, New Orleans, Louisiana 70112 USA; 2 Department of Pathology, Virginia Commonwealth University, Norfolk, Virginia 23510 USA; 3 Pathology and Laboratory Medicine Services, Southeast Louisiana Veterans Healthcare System, New Orleans, Louisiana 70112 USA; 4 Marshfield Clinic Health System, Marshfield, Wisconsin 54449 USA

**Keywords:** Acute neutrophilic vasculitis, Antigen-antibody complex, Central nervous system, Complement component, COVID-19, Karyorrhexis, Microcirculation, Microvessel, SARS-CoV-2

## Abstract

**Background:**

Hypercytokinemia, the renin-angiotensin system, hypoxia, immune dysregulation, and vasculopathy with evidence of immune-related damage are implicated in brain morbidity in COVID-19 along with a wide variety of genomic and environmental influences. There is relatively little evidence of direct SARS-CoV-2 brain infection in COVID-19 patients.

**Methods:**

Brain histopathology of 36 consecutive autopsies of patients who were RT-PCR positive for SARS-CoV-2 was studied along with findings from contemporary and pre-pandemic historical control groups. Immunostaining for serum and blood cell proteins and for complement components was employed. Microcirculatory wall complement deposition in the COVID-19 cohort was compared to historical control cases. Comparisons also included other relevant clinicopathological and microcirculatory findings in the COVID-19 cohort and control groups.

**Results:**

The COVID-19 cohort and both the contemporary and historical control groups had the same rate of hypertension, diabetes mellitus, and obesity. The COVID-19 cohort had varying amounts of acute neutrophilic vasculitis with leukocytoclasia in the microcirculation of the brain in all cases. Prominent vascular neutrophilic transmural migration was found in several cases and 25 cases had acute perivasculitis. Paravascular microhemorrhages and petechial hemorrhages (small brain parenchymal hemorrhages) had a slight tendency to be more numerous in cohort cases that displayed less acute neutrophilic vasculitis. Tissue burden of acute neutrophilic vasculitis with leukocytoclasia was the same in control cases as a group, while it was significantly higher in COVID-19 cases. Both the tissue burden of acute neutrophilic vasculitis and the activation of complement components, including membrane attack complex, were significantly higher in microcirculatory channels in COVID-19 cohort brains than in historical controls.

**Conclusions:**

Acute neutrophilic vasculitis with leukocytoclasia, acute perivasculitis, and associated paravascular blood extravasation into brain parenchyma constitute the first phase of an immune-related, acute small-vessel inflammatory condition often termed type 3 hypersensitivity vasculitis or leukocytoclastic vasculitis. There is a higher tissue burden of acute neutrophilic vasculitis and an increased level of activated complement components in microcirculatory walls in COVID-19 cases than in pre-pandemic control cases. These findings are consistent with a more extensive small-vessel immune-related vasculitis in COVID-19 cases than in control cases. The pathway(s) and mechanism for these findings are speculative.

## Background

The World Health Organization declared coronavirus disease 2019 (COVID-19) a pandemic on 11 March, 2020 [1]. COVID-19 vaccines became available in the United States by the middle of December, 2020 [2]. Severe acute respiratory syndrome coronavirus 2 (SARS-CoV-2), the viral cause of the pandemic, is the key disease factor, although each COVID-19 patient is susceptible to disease alteration through multiple disease-related, environmental, and genomic factors. These factors include severe disease, medications, mechanical ventilation, catheters, extended lengths of hospital stay [3–15], geographical [3, 16, 17] and nutritional differences [18–21], and possibly air pollutants [22].

The course of COVID-19 might also be altered by patients’ responses to secondary bacterial, viral, and/or fungal infections. These may be community acquired and/or nosocomial, and they are reported in COVID-19 patients worldwide [5, 8, 10, 13, 16, 23–53]. The most common microbial species in secondary bacterial respiratory infections in COVID-19 include members of the order *Enterobacterales* and *Staphylococcus aureus* [43]. Bacterial and viral coinfections at the time of SARS-CoV-2 diagnosis seem to be infrequent [44], while secondary infections arise commonly following hospitalization [28].

Some coinfections in COVID-19 patients, similar to findings in previous viral infections [54], may be facilitated by immune system disorders arising from the original infection. In addition, the integrity of the gut microbiome, the collective genomes of the diverse microbiota that reside in the human gastrointestinal (GI) tract, has been shown to be disturbed by SARS-CoV-2, as observed in other infectious diseases [55], causing GI dysbiosis [9, 12, 18, 20, 24, 56–62].

Alterations of the GI microbiome provide interactions that can affect the brain by influencing autoimmune-related changes through the innate immune system [9, 12, 19, 62]. A damaging host response to a viral infection is more likely to occur from a virus that can interfere with one or more of the innate immune defenses [54].

Immune-pathway alteration in a SARS-CoV-2 infection includes an excessive innate immune response [63], exemplified by an inflammatory over-activation producing cytokine storms [64]. The overacting innate system is responsible for directing an impaired adaptive host immune defense in COVID-19 patients [63], and the adaptive immune system’s response is prolonged [3]. In severe COVID-19, brain injury does not have a single pathogenic mechanism that clearly drives the dysregulation of both innate and adaptive immune system activity, which is reminiscent of autoinflammatory and autoimmune conditions. Brain damage might be caused by maladaptive host immune responses and by a bystander response that may magnify the tissue damage in COVID-19 patients [65].

Comorbid conditions in COVID-19 patients also have been associated with a severe clinical course. These include systemic hypertension, diabetes mellitus, obesity, ischemic heart disease, cancer, and a number of other conditions [66–68]. In addition to the more common COVID-19 immunomodulating comorbidities, there are less frequently encountered genomic or acquired comorbidities that might alter cytokine-related or blood-clotting systems to produce a vasculopathy. Such comorbid conditions can include rheumatoid disease, the atypical hemolytic uremic syndrome, and the antiphospholipid syndrome, among others [69–73].

The combination of such influences on the disease course, at some point during a SARS-CoV-2 infection, might factor into directing the response to the infection toward autoimmunity [65, 69, 74–79]. Specifically, autoreactivity has been noted as a feature of a severe course of COVID-19 [65, 70, 74, 79–81], just as autoimmune phenomena have been found in severe infections prior to COVID-19 [66, 82–84].

It has been proposed that severe COVID-19 is a microvascular disease [85]. Early in the COVID-19 pandemic, vascular pathology was implicated in clinical findings related to neurological effects of SARS-CoV-2 [3, 86–88], with immunosuppressive therapy showing some benefit [86]. Comorbidities such as hypertension were thought to cause at least some of the chronic vascular wall injury identified, and it was speculated that such alterations might render central nervous system (CNS) microcirculatory channels prone to autoimmune damage [89]. However, apart from chronic perivasculitis in lung [90, 91], skin [92–96], spleen [89], and heart [75, 91], there is little histopathological evidence for primary vasculitis in other organs [97].

In COVID-19 patients, histopathological evidence of a cellular inflammatory response in the brain (i.e., parenchymal infiltration of CD8^+^ T lymphocytes and of fewer CD4^+^ T cells) is at a higher level than in patients dying of severe respiratory failure, but it is at a lower level in COVID-19 patients compared to patients with long-term multiple sclerosis [98]. Focal chronic vascular wall [99, 100] and chronic perivascular inflammation are recorded in the brain in COVID-19 [99–101], while clusters of CD8^+^ lymphocytes have been identified near microglial cells in brain parenchyma [101]. Both immunoglobulin and complement component deposition involving CNS microcirculatory channels has been noted [102, 103]. These findings provide evidence of immune upregulation in COVID-19 autopsy brain parenchyma and small blood vessels.

An early theory of CNS involvement in a SARS-CoV-2 infection proposes a direct infection of CNS vascular endothelial cells. Multifocal immunostaining for SARS-CoV-2 proteins can be found in the brain [101, 102] and there is evidence of virions in brain parenchyma and in vascular endothelial cells [99, 104–107]. Some of the positive staining could be of SARS-CoV-2 spike proteins in viral fragments (pseudovirions) attached to endothelial cell receptors [108]. In a meta-analysis, 15.1% of autopsy brains are positive for SARS-CoV-2 by immunostaining [109]. The virus is only found early in the infection in most analyses although lingering infection in the brain has been documented [110].

It has been suggested that brain damage following systemic SARS-CoV-2 infection may be due to spike-protein pseudovirions (although infectious virions also could be responsible), and that the viral protein involved may drive a complement-mediated response through the lectin (alternate) complement protein pathway, resulting in microcirculatory channel wall C5b-9 (membrane attack complex) deposition [111, 112]. Lectin pathway activation in COVID-19 would suggest the possibility of immune-complex formation in COVID-19 hypersensitivity lesions [113–115]. However, C5b-9 deposition is relatively nonspecific since in the atypical hemolytic uremic and antiphospholipid antibody syndromes evidence shows that C5b-9–mediated endothelial injury can occur without significant cellular inflammation just as in the brain in COVID-19 [111]. C5b-9–mediated endothelial deposition can be more generally a sign of tissue damage such as in brain trauma [116].

Interestingly, in SARS-CoV-1, karyorrhectic polymorphonuclear neutrophils (PMNs) appear in small blood vessels in the lungs, heart, brain, and other organs [117]. Karyorrhectic PMNs forming tuft-like, beaded, or curved prongs of fragmenting nuclear material and ‘nuclear dust’ (leukocytoclasia), along with evidence of immune-complex formation shown by the presence of vascular wall complement-component deposition, are key findings in type 3 hypersensitivity vasculitis. This small-vessel vasculitis has also been designated as acute neutrophilic vasculitis, leukocytoclastic vasculitis, or hypersensitivity vasculitis [89, 93, 118–125].

Acute neutrophilic vasculitis can often be identified in the brain in hypertensive patients [118, 121, 124, 126]. It appears most often in the skin and in other organs in association with hypertension, but also in a variety of other conditions [121, 122, 125].

Patients with severe COVID-19 appear to produce IgG immune complexes that promote an inflammatory activation of PMNs. Whole blood-derived PMNs are activated more in severe than in mild COVID-19 disease, they bind IgG via Fcγ receptors and, in patients with severe COVID-19, levels of serum IgG immune complexes are higher than in healthy controls. These findings suggest that in COVID-19 IgG immune complexes might be involved in progression to severe COVID-19 disease [127].

Humoral immunity is an essential component of the adaptive immune response against SARS-CoV-2. Antibodies are produced in response to a SARS-CoV-2 infection and following a COVID-19 vaccination. In COVID-19, neutralizing immunoglobulin increases early and then a decline is found 1–6 months following symptom onset. The rise and decline in IgM, IgA, and IgG titers are offset from one another, although they have similar curves, and these periodic changes may affect immunity at different points in active COVID-19 disease [128]. Individually variable findings such as these, alone or in tandem, may allow the host’s immune system to drift into unexpected consequences, making the immune response to COVID-19 often appear to be anomalous [115].

Genomic influence might also alter the direction of such responses. In one example, *FCGR* polymorphisms can affect interactions with IgG subclasses during an infection, such as when white blood cell Fcγ receptors recognize immune complexes. These interactions might result in immune-complex deposition as an individual response to an infectious disease or in an autoimmune disease [129, 130]. This is implied by findings in COVID-19 where immune-complex formation in severe disease has been linked to Fc-receptor binding on PMNs [127].

Recent studies reveal a small impact associated with direct oral anti-coagulation medication on the development of acute neutrophilic vasculitis over a period of one day to many months after administration. These medications are commonly given to patients with atrial fibrillation and venous thromboembolism. About half the patients in which this occurs show this complication, usually in the skin, about 10 days after administration, which might be an adequate time for antigen-antibody complexes to form. Although the case rate for vasculitis development is very low, the formation of leukocytoclastic vasculitis in small skin vessels is an acute clinical event. The few medications studied adequately include heparin, warfarin, and apixaban. In addition to skin, the kidney can be affected, and initiation in these organs may be through immune-complex deposition. In one case in which leukocytoclastic vasculitis developed in the skin, a subsequent kidney biopsy revealed Henoch-Schönlein purpura [131–133]. Leukocytoclastic vasculitis, Henoch-Schönlein purpura, and related vasculitides may share similar origins [125].

## Methods

### Case selection

Following appropriate autopsy room engineering clearance and upgraded equipment acquisition, available brains removed from 36 COVID-19 autopsies were studied from patients dying in June, 2020 to December, 2021. All patients had at least one RT-PCR–positive SARS-CoV-2 test and clinical findings that were for the most part typical of COVID-19 as previously described in our region’s population [67]. The autopsy cases arrived at University Medical Center’s Emergency Department from home or by transfer to our tertiary care center from other hospitals in southeastern Louisiana or from southern Mississippi. Eight non-COVID-19 cases from 2019 were selected for immunostaining comparison of selected complement components. These eight non-COVID-19 brain cases, along with 13 other autopsy brain cases from 2019, were used as historical controls in further comparison studies. Twenty-seven contemporary control brain cases were gathered from late 2020 to late 2021.

### Mechanical ventilation, autopsy pulmonary pathology, and microbiology laboratory findings

Mechanical ventilation data were collected from electronic medical records of all cohort cases. These data were used for comparison with postmortem lung findings listed in autopsy reports.

Clinical and postmortem microbiology laboratory results including those for SARS-CoV-2 and non-SARS-CoV-2 infections were similarly collected for all case groups. For microbial findings, these lists were combined for each case in order to determine its microbial exposure.

### Statistics

Spearman’s rho was used for correlation, the Mann-Whitney *U* Test for group comparison, and chi-square (χ^2^) tests for distribution comparisons, using the 0.05 level of significance. For the evaluation of histopathological findings, data were expressed as the percentage of microscopic slides that were positive for each finding studied in each case, with this number serving as a proxy for disease burden.

## Results

### COVID-19 case cohort clinicopathological findings

Clinical features of 36 COVID-19 autopsy cases included adults ranging from 32 to 84 years of age, with 17 being male, and with 25 African Americans. Survival after hospital arrival ranged from 30 min to 84 days (mean survival, 20.4 ± 18.1 days). The patients had comorbidities typical of COVID-19 including hypertension (69.4%), diabetes mellitus (50%), obesity (50%), ischemic heart disease (41.7%), chronic pulmonary disease (22.9%), and cancer (11.1%), while dementia or mental disorder (16.7%), drug abuse (8.3%) and HIV infection (8.3%) were also represented in our cohort. Two patients had no past medical history recorded. Respiratory distress or acute respiratory failure at admission were common findings, and the major diagnosis after admission was 2019 novel coronavirus-infected pneumonia. Many patients reported headache, weakness, fatigue, or loss of smell shortly prior to admission. D-dimer serum levels were taken in 21 cases, including one within normal reference range with others varying from mildly to greatly elevated. Ventilator time varied from acute use only to multiple weeks of use, not always continuously. Steroid therapy was provided in 22 cases, remdesivir was given in 13 cases, and there was administration of tocilizumab in four cases (administered once, from two to 30 days prior to patient demise) and of convalescent plasma in one case. One patient was fully vaccinated against SARS-CoV-2. Two patients had one vaccine dose.

There were seven deaths between 5 and 9 AM (19.4% of cases, a period accounting for 16.6% of the day) and 11 deaths between 4 and 10 AM (30.6% of cases; 25% of the day). Compared to the remainder of the cohort, for deaths between 5 and 9 AM (χ^2^ = 0.12; *p* > 0.7) and for deaths between 4 and 10 AM (χ^2^ = 0.4; *p* > 0.5), there was no significant difference (Table 1). The postmortem interval between death and autopsy for the COVID-19 cohort cases had a mean time of 2.2 days (median, 1.1 days; range 0.25–11 days).

**Table 1:** Selected clinical data for 36 COVID-19 autopsy brain cases

Case no.	Age (yr)/Sex	Major comorbidities	BMI^a^	D-dimer^b^	Ventilator (da)	COVID-19–related treatment	Survival (da)/time of death (hr)
1^c, d^	67/M	DM2	20.1	N/R	Acutely	N/R	0.021/0530
2^c^	61/F	N/R	N/R	N/R	Acutely	N/R	0.035/1316
3	44/M	HIV, HCV, TB, head injury several months previously	25.1	N/R	Acutely	N/R	0.063/0543
4	84/F	HTN, IHD, arthritis, dementia, chronic venous stasis	23.4	N/R	Acutely	N/R	0.09/1408
5	47/F	HTN, DM	N/R	N/R	Acutely	N/R	0.167/1728
6	72/F	Obesity, influenza A	43	N/R	Acutely	N/R	0.25/1245
7	49/M	HTN, obesity, SAD, BD	34.9	6.9 FEU	1	N/R	1/1404
8	61/M	HTN, IHD, COPD, ethanol abuse	22	620 DDU	2	DEX, REM	4/0511
9	76/F	HTN, DM, IHD, HIV	25.9	326 DDU	None	DEX	5/0444
10^c^	37/M	HTN, HCV, HPV, hepatocellular carcinoma, SAD	26	N/R	None	N/R	5/0744
11^c^	49/F	HTN, obesity	67.2	N/R	3	DEX, TOC	8/2340
12	61/F	IHD, COPD, emphysema, asthma, rectal carcinoma	22.7	167 DDU	8	DEX	8/0243
13	64/F	HTN, DM2, old stroke, obesity	34.6	N/R	2	N/R	10/1350
14	65/M	HTN, DM, IHD, COPD, pulmonary fibrosis, obesity	32.6	2551 DDU	< 1	DEX, REM	10/1330
15	63/M	HTN, atrial fibrillation, obesity	46.1	45144 DDU	12	DEX, VAN	12/2025
16^c^	53/F	HTN, DM2, COPD, pulmonary hypertension, emphysema, obesity	47	N/R	None	TOC	13/1818
17^c^	58/F	HTN, DM, IHD, obesity, atrial fibrillation, CRD	52	N/R	5	None	16/1749
18	61/F	HTN, DM2, obesity	51.4	2528 DDU	17	DEX	17/1957
19	68/M	HTN, DM2, CRD, anemia of chronic disease	21.8	2574 DDU	None	DEX	19/1100
20	62/F	HTN, DM2, IHD, bilateral carotid artery stenosis, obesity, HCV, hypothyroidism	36.8	523 DDU	5	DEX, REM	19/0437
21	69/F	HTN, DM2, IHD, obesity, CRD	44.5	2462 DDU	11	DEX, REM	22/0708
22^e^	56/F	HTN, DM, IHD, COPD, asthma, obesity, blood clotting disorder, DVT, liver disease, CRD	45.4	N/R	None	DEX, REM	23/0914
23	73/F	DM2	29	2805 DDU	7	DEX, REM, TOC	24/1033
24	59/F	IHD, remote head trauma, small recent and intermediate cerebral infarcts, small intermediate/remote cerebellar infarcts	21.1	51317 DDU	None	DEX	26/0248
25	70/M	HTN, DM2, IHD, obesity, COPD, asbestosis, obesity, CRD	77.0	527 DDU	23	DEX, REM	27/0322
26	61/M	N/R	20.7	8.07 FEU	28	DEX, REM	29/0423
27	63/M	HTN, DM2, IHD, SAD, HBV, HCV, schizophrenia, BD, hepatic cirrhosis	21.4	N/R	2, later 5, later 9	N/R	29/1654
28^c^	66/M	HTN, HBV, hepatic cirrhosis	28.3	2884 DDU	13	DEX, REM	33/2108
29^e^	63/M	HTN, DM, IHD, COPD, asbestosis, TTRA, HIV, HCV, recent left-sided weakness, CRD, hepatic cirrhosis	21.8	1450 DDU	None	N/R	35/2345
30	34/F	DM, obesity	58.5	N/R	10, then 20	N/R	36/2159
31^e^	62/M	HTN, DM2, DVT, BD, seizure history	24.8	21604 DDU	13, later 15	DEX, REM	39/1514
32	66/F	HTN, obesity, schizophrenia, gout	40.2	252 DDU	18	DEX, REM, TOC	39/0724
33	64/F	COPD, obesity, colon cancer	36	0.62 FEU	24	DEX, REM	41/1800
34	52/F	HTN, IHD, obesity	54.6	5507 DDU	18	DEX	44/1138
35	32/M	DM	23.1	N/R	44	DEX, REM	56/0717
36	64/F	HTN, obesity, schizophrenia, ulcerative colitis	33.3	962 DDU	2, then 32	DEX, CP	84/2152

Case data are arranged in order of increasing time of survival (days)

*BMI* Body mass index; *DM2* DM type 2; *N/R* None recorded; *HIV* Human immunodeficiency virus infection; *HCV* Hepatitis C virus infection; *TB* Tuberculosis; *HTN* Systemic hypertension; *IHD* Ischemic heart disease; *DM* Diabetes mellitus untyped; *COPD* Chronic obstructive pulmonary disease; *DEX* Dexamethasone; *REM* Remdesivir; *SAD* Substance abuse disorder; *BD* Bipolar disorder; *HPV* Human papillomavirus infection; *IV* Intravenous; *TOC* Tocilizumab; *CRD* Chronic renal disease; *DVT* Deep vein thrombosis; *HBV* Hepatitis B virus infection; *TTRA* Transthyretin amyloidosis; *CP* Convalescent plasma

^a^BMI ratio (kg/m^2^) is 18.5 to 24.9 in most normal adults; obesity, ≥ 30

^b^D-dimer testing uses either the D-dimer unit reference range (normal, < 250 ng/mL DDU) or the fibrinogen-related reference range (normal, < 0.5 mg/L FEU)

^c^SARS-CoV-2 delta variant (PCR analysis)

^d^Received two doses of vaccine against SARS-CoV-2

^e^Received one dose of vaccine against SARS-CoV-2

Formalin-fixed, paraffin-embedded tissue included 32 to 50 blocks from cerebrum, cerebellum, and brainstem in each COVID-19 cohort case. These 1,284 blocks included all cerebral and cerebellar lobes and all three brainstem levels. Olfactory bulbs and tracts were taken in 27 cases. All microscopic sections were studied with hematoxylin-eosin staining, with immunostaining for white blood cell and serum proteins on selected cases.

Gross examination of the COVID-19 cohort included brain swelling with cerebellar tonsillar herniation in three cases, non-hemorrhagic cerebral infarcts in eight cases, and mild signs of brain atrophy consistent with age and comorbidities in a few cases. Three cases had significant numbers of petechial hemorrhages scattered in the brain, while several cases had only an occasional petechial hemorrhage. The most significant hemorrhage was in Case 4 where histopathological findings associated with large hemorrhages included recent cerebral infarcts. Both the hemorrhages and infarcts in Case 4 contained significant infiltrations of PMNs. Stains were negative for micro-organisms. One of the cases with cerebellar tonsillar herniations had global hypoxic nerve-cell change. Case 26 had large, non-hemorrhagic intermediate infarcts in the territory of both middle cerebral arteries. Thrombotic or atherosclerotic obstruction of the common carotid arteries had been identified in that case by clinical imaging.

Histopathologically, scattered microcirculatory perivascular hemorrhages were identified in 25 of 36 cases (69.4%), and when found they appeared in up to 60% (mean, 12.2 ± 2.3%) of tissue blocks. Paravascular microhemorrhages and a few petechial hemorrhages (together, considered to be small parenchymal hemorrhages) were present in 13 cases, involving up to 25.7% (mean, 3.42 ± 1.2%) of blocks. Eight cases had both perivascular hemorrhages and small parenchymal hemorrhages.

Microthrombi were frequently present in the microcirculation throughout the cohort although in most instances they did not appear to be obstructive. Immunostaining for platelet protein or for fibrin or fibrinogen showed very few microvascular lumina with apparent obstruction. Case 15, with hypertension and atrial fibrillation, had relatively few non-microvascular findings, but a non-occlusive organizing basilar artery thromboembolus was present. Thirteen cases (36.1%) had microglial nodules, mostly in the brainstem. Isolated neuronophagia was present in the brainstem in six cases (16.7%).

**Fig. 1:**
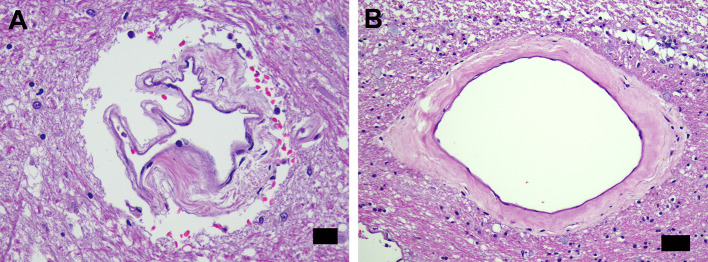
Cerebral microvasculopathy with mural collagenosis. (**A**) Severe mural distortion with irregular adventitial collagenosis of a microvessel in midbrain tegmentum near substantia nigra (Case 13). (**B**) Dilated microvessel with somewhat compact adventitial collagenosis in periventricular calcarine white matter (Case 12). Scale bars: 20 μm in (**A**); 50 μm in (**B**)

The most numerous brain findings in this cohort, aside from frequent neuronal hypoxic change, were in the microcirculation. The most common microcirculatory alteration was simple dilation (ectasia). In all but one case, variable numbers of microcirculatory channels had adventitial collagenosis or more compact hyaline sclerosis (Fig. 1). The third type of reactive microvasculopathy was intussusceptive arborization (IA), often seen as ‘mini-glomeruloid’ formations that were found in 15/36 (41.7%) cases.

**Fig. 2:**
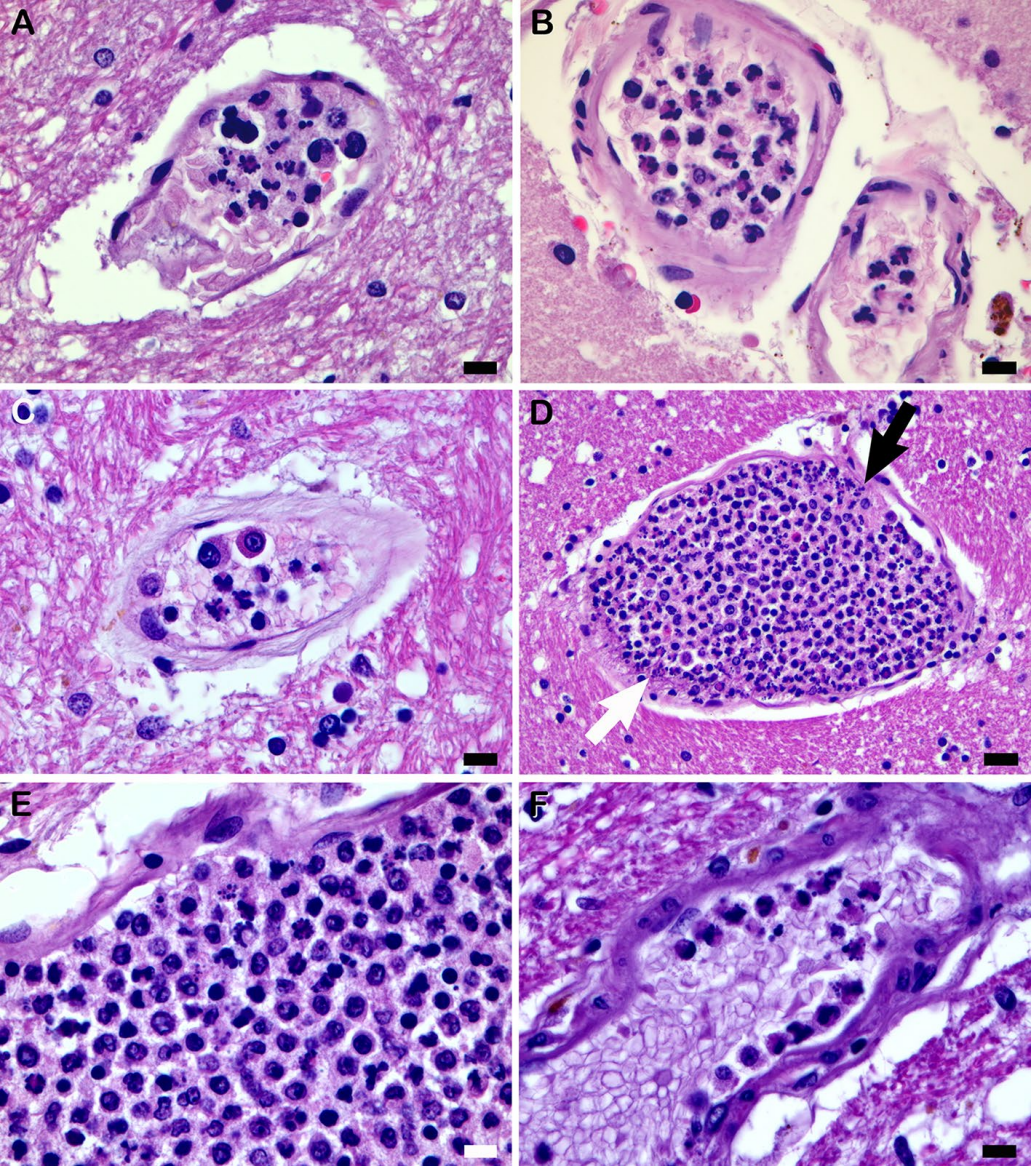
Acute endotheliitis. (**A**) In midbrain tegmentum, a thin-walled microcirculatory channel has many karyorrhectic PMNs including some fragmenting into dot-like nuclear dust (Case 10). (**B**) Similar finding as in (**A**) is seen here in subarachnoidal microvessels between folia of the cerebellar superior vermis (Case 10). (**C**) Small microcirculatory channel with mural collagenosis in medial temporal subependymal white matter has intraluminal karyorrhectic PMNs and mononuclear cells (Case 14). (**D**) In lateral temporal white matter, a dilated thin-walled microvessel is filled with PMNs, many with karyorrhexis, and mononuclear cells. There is scattered ‘nuclear dust’ (black arrow) and a few karyorrhectic PMNs appear to be transmigrating into fibrous adventitia (white arrow) (Case 14). (**E**) Mixture of karyorrhectic PMNs, some ‘nuclear dust’, and many mononuclear cells in very dilated microvessel in internal capsule near hypothalamus (Case 23). (**F**) Pyknotic and karyorrhectic PMNs arrayed along luminal border of microvessel in lateral hypothalamus (Case 34). Scale bars: 10 μm in (**A**–**C**, **E** and **F**); 20 μm in (**D**)

All 36 cases had acute neutrophilic vasculitis with a variable amount and distribution within the cohort from frontal lobe to medulla. This finding included only a few vessels in three cases. Acute neutrophilic vasculitis was recognized in the brain’s microcirculation by the presence of intraluminal PMNs with karyorrhexis (nuclear fragmentation, often including ‘nuclear dust’ formation). Affected microcirculatory channels generally had a relatively thin wall, including some large microvessels (Fig. 2) (Table 2). In our COVID-19 cohort, acute neutrophilic vasculitis with leukocytoclasia was present focally or multifocally in 37.8 ± 26.6% of 1,284 microscopic slides of brain, representing a significant disease burden in most of the cases.

**Table 2:** Vascular sites of polymorphonuclear nuclear (PMN) karyorrhexis and acute perivasculitis in 36 COVID-19 autopsy brains

Case no.	CNS microcirculatory intravascular PMN karyorrhexis/acute endotheliitis	Acute perivasculitis
1	L thalamus, L int capsule	None
2	L frontal lobe, parietal lobes, R lat temp lobe, R insula, L calcarine cortex, L med hypothalamus, R int capsule, R lent nuc, cblr inf vermis, rostral and mid-level pons	Mid-level pons
3	Cblr inf vermis, R midbrain teg, L pontine teg, bulbar nuc of spinal tract of nerve V, nuc cuneatus	None
4	R occipital lobe, L basal ganglia, rostral R midbrain, caudal L midbrain, rostral pons, mid pons, caudal pons	Rostral L midbrain teg
5	R parietal lobe, med and lat temp lobes, R insula, L calcarine cortex, R and L corpus striatum, R thalamus, cblr R lat lobe and vermis, rostral L pons	None
6	Occipital lobe, putamen, thalami, anterior perforated substance, cblr lat lobes, midbrain and pontine teg levels, basis pontis and bulbar levels	R temp fusiform gyrus
7	R lat temp lobe, R int capsule, cblr white matter, R pontine teg, L pontine locus coeruleus, multiple basis pontis levels	Cblr L lat lobe
8	L frontal lobe, white matter adjacent to tail of caudate nucleus, R hippocampus, cblr R lat lobe	None
9	R frontal lobe, L temp entorhinal cortex, R lat temp lobe, R parietal lobe, calcarine cortex, R thalamus, R putamen, int capsule, midbrain tectum, caudal midbrain, rostral basis pontis, substantia reticularis by inf olivary nucleus	R substantia nigra, caudal midbrain teg, R basis pontis
10	L frontal lobe; parietal lobes; L med temp lobe; R and L insula; L corpus striatum; thalami; lent nuclei; cblr lat lobes and vermis; rostral, mid-level, and caudal midbrain and pons; mid-level medulla	R lat temp lobe, R insula, L lent nuc, mid-level midbrain, pons, medulla
11	Parietal lobes, R and L insula, R and L corpus striatum, lenticular nuclei, thalami, int capsules, R rostral midbrain, L mid-level and caudal midbrain	L frontal lobe, L insula, L int capsule, L mid-level midbrain
12	R frontal lobe; lat and med temp lobes; R and L insula; L calcarine cortex; R and L corpus striatum; lent nuclei; thalami; cblr lat lobes and vermis; rostral, mid-level, and caudal midbrain and pons; rostral and mid-level medulla	Rostral pons
13	L frontal lobe, R calcarine cortex, L int capsule, cblr R lat lobe, mid-level midbrain, mid-level pons	None
14	Frontal, parietal, temp, insular, and calcarine cortex; R hippocampus; lent nuclei; thalami; caudate nuclei; int capsules; L external and extreme capsules; cblr lat lobes and vermis; middle cblr peduncle; midbrain; pontine teg and basis pontis; nuc of tractus solitarius area; nuclei of cranial nerves V and XII; inf vestibular nuclei; inf olivary nuclei; olivocerebellar tracts; pyramid	L globus pallidus interna
15	Frontal lobes, parietal lobes, R temp entorhinal cortex, L lat temp lobe, occipital lobes, R putamen, thalami, L basal forebrain, cblr R lat lobe, rostral and caudal midbrain, basis pontis	Rostral L basis pontis
16	Int capsules, R putamen, R thalamus	L putamen
17	L parietal lobe, R calcarine cortex, R corpus striatum, R int capsule	None
18	R frontal lobe, L parietal lobe	R lat temp lobe
19	R frontal lobe, L lat temp lobe, R thalamus, midbrain, mid-level and caudal basis pontis	None
20	R frontal orbital cortex; R lat temp lobe; L parietal lobe; L int capsule; cblr R lat lobe, dentate nuc, and middle cblr peduncle; caudal pons; rostral medulla; caudal bulbar pyramidal tract	R frontal orbital cortex, R thalamus, L lent nuc, L hypothalamus, rostral and caudal midbrain, pons
21	Frontal lobes, R parietal lobe, lat temp lobes, R med temp lobe, L int capsule, cblr L lat lobe, mid-level pons, mid-level medulla	Parietal lobes, R med temp lobe, L corpus striatum, R putamen, mid-level medulla
22	R frontal lobe; R lat temp lobe; L lat and med temp lobe; R and L insula; L thalamus; R int capsule; cblr lat lobes and vermis; caudal L midbrain; middle cblr peduncle; rostral, mid-level, and caudal pons and medulla	L lent nuc
23	Parietal lobes; R entorhinal cortex; R lat temp lobe; L insula; L calcarine cortex; R and L corpus striatum; thalami; cblr L lat lobe and vermis; caudal midbrain; rostral, mid-level, and caudal pons; mid-level and caudal medulla	R int capsule, cblr R lat lobe and vermis, mid- level pons
24	R med temp lobe, R putamen, mid-level pons	None
25	L parietal lobe, R lat and med temporal lobe, R caudate nuc, putamina, R thalamus, R midbrain teg and bilateral crus cerebri, L pontine teg, basis pontis	Rostral R midbrain, multiple L pontine levels
26	Cblr vermis, caudal pontine teg	None
27	L frontal lobe, L insula, R parietal lobe, cblr lat lobes and vermis, rostral R midbrain, mid-level pons, rostral medulla	None
28	L frontal lobe, L lat temp lobe, R and L insula, parietal lobes, R and L corpus striatum, lent nuclei, R claustrum, L thalamus, int capsules, cblr lat lobes, L middle cblr peduncle, R and L mid-level midbrain, rostral pontine teg and basis pontis, mid-level R and L basis pontis, caudal basis pontis, caudal medulla	R hypothalamus
29	L lat temp lobe, L insula, R calcarine cortex, head of L caudate nuc, rostral R pontine teg, caudal L pontine teg near superior vestibular nuc, mid-level medulla near lat reticular nuc	R supraoptic nuc
30	R parietal lobe, R calcarine cortex, L thalamus, rostral bulbar inf olivary nuc	R parietal lobe
31	Frontal lobes; L parietal lobe; L insula; L claustrum; extreme capsules; L corpus striatum; L globus pallidus; cblr L lat lobe and vermis; rostral, mid-level, and caudal pons; mid-level medulla	R frontal lobe, cblr lat lobes, rostral midbrain, mid-level and caudal pons, mid-level medulla
32	R frontal lobe, L parietal lobe, R corpus striatum, posterior thalami, cblr L lat lobe	R frontal lobe, R lent nuc, caudal L midbrain, rostral and mid-level pons
33	R frontal lobe, L temp and parietal lobes, L thalamus, L putamen, globus pallidus externa, cblr lat lobes, rostral and caudal R midbrain teg and substantia nigra, rostral L substantia nigra, caudal R crus cerebri, rostral R basis pontis, pyramidal tract	None
34	L frontal lobe; parietal lobes; R lat and med temp lobe; L lat temp lobe; R and L insula; R and L calcarine cortex; R and L corpus striatum; R hypothalamus; int capsules; cblr lat lobes and vermis; rostral L midbrain; mid-level midbrain crus cerebri and substantia nigra; caudal midbrain; rostral, mid-level, and caudal pons and medulla	Rostral L midbrain, mid- level R and L midbrain, inf cblr peduncle, caudal medulla
35	Frontal lobes, R parietal lobe, lat temp lobes, R and L insula, R and L corpus striatum, R lent nucleus, L thalamus, cblr vermis, caudal midbrain, mid-level and caudal pons	R basis pontis
36	L frontal lobe, R and L lateral temp lobes, R hippocampus, R parietal lobe, R ant comm, R thalamus, R lent nuc, L globus pallidus, internal capsules, R hypothalamus, cblr lat lobes, right substantia nigra, caudal crus cerebri, R rostral basis pontis, R and L mid-level and caudal basis pontis, R and L caudal pontine teg, bulbar reticular formation	L lat temp lobe, R midbrain teg

Abbreviations: ant, anterior; cblr, cerebellar; comm, commissure; inf, inferior; int, internal; lat, lateral; L, left; lent, lenticular; med, medial; MLF, medial longitudinal fasciculus; nuc, nucleus; R, right; teg, tegmentum; temp, temporal

**Fig. 3:**
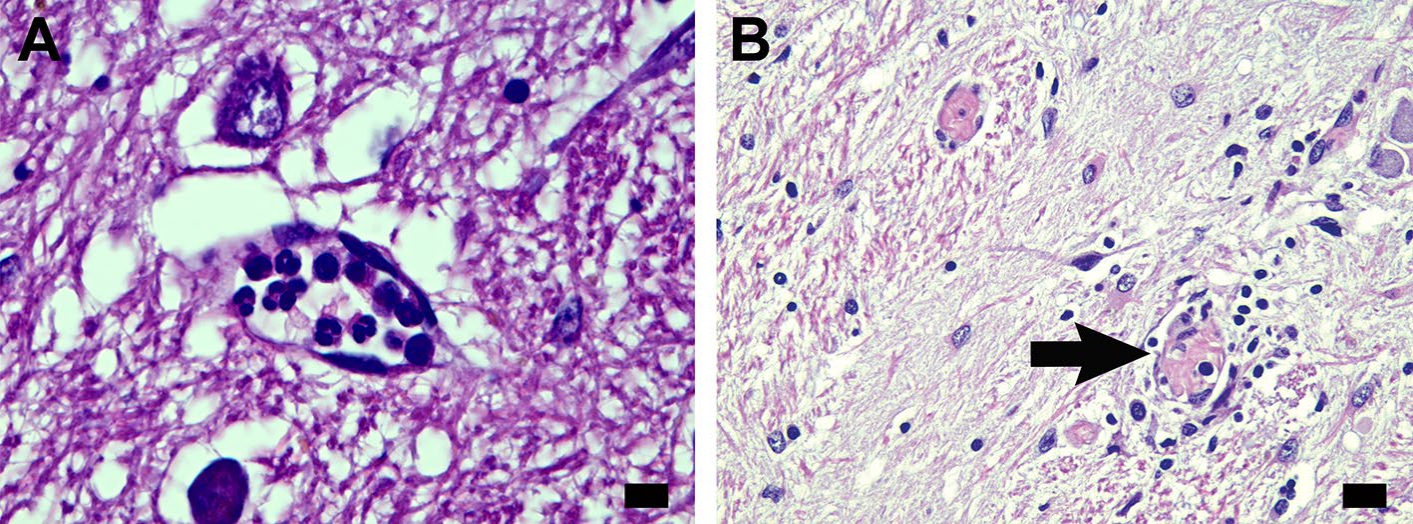
Nucleus of the tractus solitarius region. (**A**) Small, dilated microcirculatory channel adjacent to the nucleus in the rostral medulla contains PMNs with karyorrhectic nuclei (Case 14). (**B**) There is a thromboembolized microcirculatory channel at edge of nucleus of tractus solitarius (arrow) and similar blood vessel near the nucleus, with prominent reactive gliosis in the field (Case 36). Scale bars: 10 μm in (**A**); 20 μm in (**B**)

A small, dilated microcirculatory channel in the nucleus of the tractus solitarius contained karyorrhectic PMNs (Fig. 3A). Two microcirculatory channels with thromboemboli on routine hematoxylin-eosin stain were of note because of their location at the edge of and near the nucleus of the tractus solitarius. In this instance, the thromboemboli were accompanied by prominent reactive astrocytosis around the vascular channels (Fig. 3B).

In comparing acute neutrophilic vasculitis with small hemorrhagic vessel-associated findings, there was a very weak correlation between the vasculitis and the combined group of perivascular hemorrhages, paravascular microhemorrhages, and petechial hemorrhages (Spearman’s rho = 0.097). The perivascular hemorrhages, when compared only to the group of small parenchymal hemorrhages, had even less correlation (Spearman, *r* = 0.08). There was a very weak correlation between the vasculitis and perivascular hemorrhages alone (Spearman, *r* = 0.12). Interestingly, all of the small parenchymal hemorrhages as a group tended to have a relatively low disease burden in cases with a high burden of acute neutrophilic vasculitis as shown by a weak negative correlation (Spearman, *r* = -0.21). When comparing survival with the total burden of small parenchymal hemorrhages in our cohort, the relationship was not significant (Mann-Whitney *U*-Test, z-ratio = 0.49, *p* > 0.6; Spearman, *r* = 0.06).

Significant direct microcirculatory mural damage from acute neutrophilic vasculitis was not often found histopathologically, although some ectasia was generally noted. In 15 cases (41.7%) at least one dehiscent capillary (or possibly larger disrupted thin-walled microvessel) was observed, with or without limited perivascular hemorrhage and generally without karyorrhectic PMNs or ‘nuclear dust’.

In most of the cohort cases, there were a few scattered microcirculatory vessels with a suggestion of poorly-formed platelet-fibrin thrombi accompanied by PMNs and a few mononuclear cells, and karyorrhectic PMNs were in a few of these vessels (Fig. 2C). Intraluminal eosinophils were found occasionally. Fibrinoid necrosis and intimal fibrous organization were not seen in the microcirculation.

**Fig. 4:**
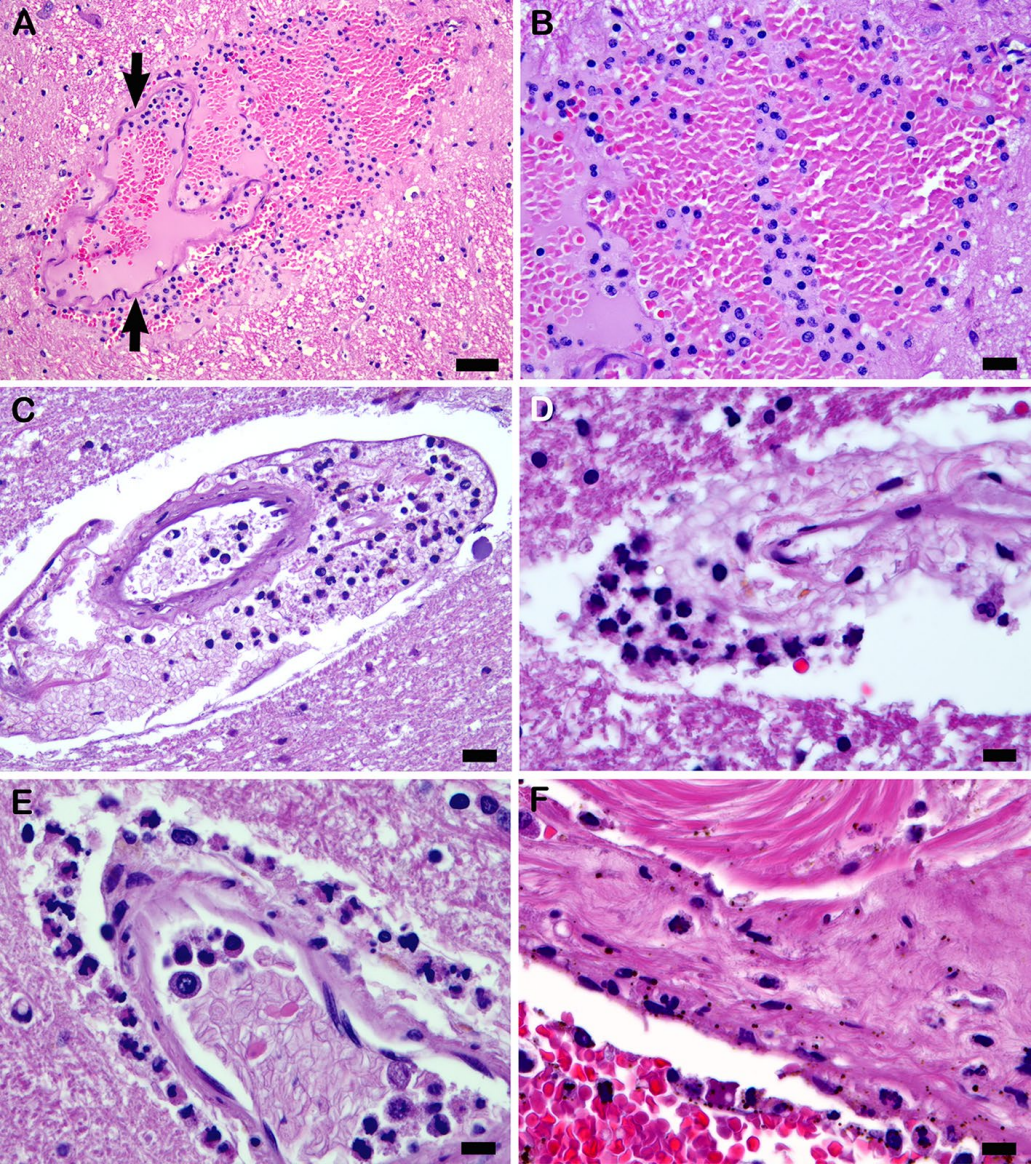
Acute perivasculitis and mural PMN transmigration. (**A**) Dilated, thin-walled microvessel with serpiginous profile (arrows) is surrounded by perivascular hemorrhage containing PMNs in rostral pontine tegmentum (Case 25). (**B**) Higher magnification of perivascular hemorrhage in **A** includes many PMNs indicating acute perivasculitis (Case 25). (**C**) Cerebellar folial white matter microvessel with collagenosis and perivascular hemorrhage with PMNs (Case 31). (**D**) Temporal fusiform gyrus white matter microvessel has perivascular hemorrhage with PMNs, some appearing to be karyorrhectic (Case 31). (**E**) Many PMNs ringing microvessel in mid-level basis pontis are karyorrhectic (Case 10). (**F**) Subarachnoidal arteriolar wall with transmigrating PMNs (Case 31). Scale bars: 50 μm in (**A**); 20 μm in (**B**) and (**C**); 10 μm in (**D**–**F**)

Transmural PMN migration that resulted in acute perivasculitis involved at least one microvessel in 25 cases (69.4%) (Fig. 4A–E) (Table 2). Karyorrhectic PMNs were often identified among the perivascular PMNs of acute perivasculitis (Fig. 4C–E). Mural transmigration of PMNs from the lumen to the perivascular space was apparent in a few dilated, thin-walled microvessels (Fig. 2D). Four cases had a few subarachnoidal arterioles in which transmigration was more prominently seen (Fig. 4F). In two of these cases, arteriolar transmigrating PMNs were immunostained for myeloperoxidase.

Most cohort cases had at least a few small blood vessels with mild perivascular chronic inflammatory cellular infiltration that may have represented immunosurveillance. Increasingly prominent yet not marked focal perivascular cuffing appeared to represent chronic perivasculitis in at least four or five cases. Immunostaining showed more CD8^+^ T cells than CD4^+^ T cells in these inflammatory deposits, and relatively few B cells were present.

### Contemporary and historical control cases compared to COVID-19 cohort cases

Among the contemporary control cases, nine were vaccinated for COVID-19, including one of six patients with resolved COVID-19. There was no difference in the rate of acute neutrophilic vasculitis in the brain at autopsy between contemporary controls receiving or not having a record of receiving any COVID-19 vaccine (χ^2^ = 0.5; *p* > 0.4). The six resolved COVID-19 cases died from 2 to 15 months (mean, 8.5 months) after symptom onset. The only case of resolved COVID-19 with acute neutrophilic vasculitis with leukocytoclasia had this finding in multiple midbrain microcirculatory channels. This patient also had positive cultures for *Streptococcus agalactiae*, *Enterobacter cloacae* complex, and *Achromobacter xylosoxidans* (Table 3). The mean postmortem interval for the 21 non-COVID-19–related contemporary control cases was 1.5 days (median, 1 day; range, 0.25–7.4 days). For the six contemporary controls with resolving COVID-19, the mean postmortem interval was 2 days (median, 1.6 days; range, 1–3.5 days).

**Table 3:** Contemporary control autopsy brain cases

Case no.	Age (yr)/Sex	Major clinical and autopsy diagnoses; positive blood cultures	Ventilator use (da)	Anti-coagulation treatment	Karyorrhectic CNS small blood vessels	Hospital survival (da)
	**Non-COVID-19 cases**					
1	67/F	HTN, DM, COPD, obesity, cancer	N/R	Aspirin, heparin, enoxaparin	No	5
2	34/F	SAD, acute pancreatitis, hepatic cirrhosis	Acutely	N/R	Yes	0.03
3	54/M	HTN, DM, CRD, clinical sepsis, necrotizing pneumonia	4	N/R	Yes	4
4	67/F	DM2, obesity, cancer, acute *Candida albicans* pneumonia	Acutely	Heparin	No	0.25
5	61/M	HTN, DM2, IHD, SAD	Acutely	Aspirin	No	0.06
6	60/F	HTN, IHD, obesity, DVT, PE, retroperitoneal abscess (*Streptococcus anginosus*), transverse colectomy, small bowel resection	E/P	Heparin, apixaban	Yes	3
7	47/M	HTN, IHD, obesity, atrial fibrillation; *Klebsiella pneumoniae*	Acutely	Heparin	Yes	0.04
8^a^	64/M	HTN, DM, IHD, cancer, CRD	Acutely	Heparin	Yes	0.04
9	59/M	HTN, DM, obesity, CRD, PE	3.5	Aspirin, heparin	No	3.5
10^b^	55/F	HTN, DM, IHD, SAD, hepatic cirrhosis	Acutely	N/R	Yes	9
11	71/M	HTN, COPD, GI ulcer hemorrhages; *Clostridium innocuum*, *Bacteriodes uniformis*, *Lactobacillus* spp.	Acutely	N/R	Yes	0.4
12^a, c^	64/M	HTN, IHD, SSD, MDS	Acutely	Aspirin, apixaban	Yes	9
13^a^	67/F	HTN, IHD, COPD, obesity, CRD, diffuse cerebral atrophy; *Citrobacter braakii*, *Serracia marcesens*, *Klebsiella pneumoniae*	N/R	N/R	No	13
14^a^	61/M	HTN, DM2, IHD, HCV, CRD, hepatic cirrhosis, small remote cerebellar infarct	1	Aspirin, clopidogrel	Yes	1.5
15	62/F	HTN, obesity, SAD, hepatic cirrhosis, toxic epidermal necrosis, diffuse cerebral atrophy; *Staphylococcus aureus*	N/R	N/R	No	5
16^a^	49/M	IHD, obesity, gastric necrosis, intestinal gangrene, recent globus pallidus infarcts; *Enterococcus faecalis*, *Escherichia coli*, *Enterobacter cloacae* complex, *Bacteroides thetaiotaomicron*, *Clostridium sporogenes*	E/P	Heparin	Yes	2
17	78/F	IHD, non-COVID ARDS, obesity, DVT, PE	4	Heparin, apixaban, enoxaparin	Yes	9.5
18	43/M	HTN, asthma, obesity, bronchial mucous plugs; *Escherichia coli*	Acutely	N/R	No	0.04
19^a^	48/M	HTN, DM2, IHD, obesity, SAD, acute bronchopneumonia	3	Heparin, enoxaparin	No	3
20	35/F	HTN, obesity, post-partum presumptive viral meningoencephalitis, pulmonary artery saddle embolus	N/R	N/R	No	0.5
21^a^	44/M	HTN, DM1, cancer, small corpus striatum resolving infarct; *E. faecalis*, *E. faecium*, *E. casseliflavus*, *K. pneumoniae*	N/R	N/R	No	7
	**Resolved COVID-19 cases/post-COVID survival time (mo.)**					
22	74/M	HTN, DM, IHD, renal failure, LUE AVF severe hemorrhage/9	N/R	N/R	No	0.03
23	76/M	HTN, IHD, atrial fibrillation, SAD, CRD/10	3	Apixaban	No	3
24	43/M	SAD, HCV, hepatic cirrhosis, CML with brain metastases/2	0.5	N/R	No	5
25	60/F	HTN, IHD, SAD, ruptured aortic aneurysm/5	N/R	Heparin	No	9
26^b^	64/F	HTN, IHD, COPD, obesity, cancer/15; *Staphylococcus aureus*, *Achromobacter xylosoxidans*	None	Heparin	No	0.25
27	69/M	HTN, IHD, atrial fibrillation, obesity/10; *Streptococcus agalactiae*, *Enterobacter cloacae* complex	N/R	Enoxaparin	Yes	11

Contemporary case data in order of autopsy case accession selected for age and comorbidities (cases 1–21) or for history of resolved COVID-19 (cases 22–27), Fall 2020 through Fall 2021

Ethnicity: African American = 21; Caucasian = 6

Abbreviations: HTN, systemic hypertension; DM, diabetes mellitus untyped; COPD, chronic obstructive pulmonary disease; N/R, none recorded; SAD, substance abuse disorder; CRD, chronic renal disease; DM2, DM type 2; IHD, ischemic heart disease; DVT, deep vein thrombosis; PE, pulmonary embolism; E/P, episodic/procedural; GI, gastrointestinal; SSD, sickle cell disease; MDS, myelodysplastic syndrome; HCV, hepatitis C virus infection; ARDS, acute respiratory distress syndrome; DM1, DM type 1; LUE AVF, left upper extremity arteriovenous fistula

^a^Received two doses of vaccine against SARS-CoV-2

^b^Received one doses of vaccine against SARS-CoV-2

^c^Blood anti-neutrophil cytoplasmic antibody (ANCA) pattern not detected by immunofluorescence microscopy; complement components 3 and 4 within reference range

For contemporary controls, 21/27 cases (77.8%) had hypertension, 12/27 cases (44.4%) had acute neutrophilic vasculitis, and 9/27 cases (33.3%) had both hypertension and acute neutrophilic vasculitis. The microcirculatory channels with karyorrhectic PMNs in contemporary controls were focal or multifocal and included cerebrum (seven cases), cerebellum (five cases), and brainstem (four cases).

In historical controls, 15/21 cases (71.4%) had hypertension, 8/21 cases (38.1%) had acute neutrophilic vasculitis with karyorrhectic PMNs in one or a few microcirculatory channels, and 6/21 cases (28.6%) had both hypertension and acute neutrophilic vasculitis (Table 4). The karyorrhectic PMNs in historical controls involved focal or multifocal microcirculatory channels in the cerebrum (six cases), cerebellum (one case), and brainstem (three cases). In the historical control cases, the postmortem interval was 1.5 days (median, 1.3 days; range, 0.2–3.8 days).

**Table 4:** Historical control autopsy brain cases

Case no.	Age (yr)/Sex	Major clinical and autopsy diagnoses; positive blood cultures	Ventilator use (da)	Anti-coagulation treatment	Karyorrhectic CNS small blood vessels	Hospital survival (da)
1	61/M	HTN, DM2, obesity, DLE, lung abscess; diphtheroids	Acutely	Enoxaparin	Yes	3
2	64/F	HTN, DM, COPD, IHD, obesity, cancer; *Acinetobacter baumannnii*, diphtheroids	2	Enoxaparin	No	2
3	70/F	HTN, obesity, clinical remote stroke; *Klebsiella pneumoniae*	N/R	Warfarin, enoxaparin	Yes	4
4	53/F	HTN, DM2, IHD, obesity; *Clostridium perfringens*, α-hemolytic *Streptococcus*	Acutely	N/R	Yes	0.2
5	66/F	HTN, DM, IHD, obesity, remote stroke NCH; *Enterococcus faecalis*, *Pseudomonas mirabilis*	N/R	Aspirin, apixaban	Yes	1
6	52/M	HTN, DM, IHD, CRD, pancreatitis; *Klebsiella pneumoniae*	Acutely	N/R	No	9
7	61/F	HTN, obesity, cancer, PE; *Enterococcus gallinarum*	E/P	Heparin, enoxaparin	Yes	11
8	69/F	HTN, BD, basal ganglia hemorrhage; *K. pneumoniae*	Acutely	N/R	No	0.2
9	65/F	HTN, COPD, focal frontal lobe perivascular brain hemorrhage; diphtheroids	Acutely	Heparin	No	0.04
10	57/M	DM2, PE	Acutely	Enoxaparin, tPA	No	2
11	35/M	Obesity, epilepsy, frontotemporal FCD	Acutely	N/R	No	0.04
12	38/M	Obesity, aortic dissection, PE	N/R	Heparin, tPA	Yes	12
13	39/M	HCV, Lyme disease, SAD, CAH, cervical spinal epidural abscesses; *Staphylococcus aureus*	N/R	N/R	Yes	6
14	47/F	HTN, DM2, IHD, COPD, obesity, congenital heart disease	N/R	tPA	No	0.6
15	86/F	HTN, cancer, CRD, aortic dissection, small remote basal ganglia infarcts; *Staphylococcus aureus*, α-hemolytic *Streptococcus*	Acutely	N/R	No	0.02
16	91/F	HTN, IHD, CRD, BD, schizophrenia, stroke NCH; *K*. *pneumoniae*, *Escherichia coli*, *Clostridium perfringens*	Acutely	N/R	Yes	0.04
17	63/M	HTN, IHD, BD, DVT, PE, 0.5-cm left frontal remote infarct	E/P	Enoxaparin	Yes	11
18	59/F	HTN, arthritis, gastric ulcers, sepsis, intestinal and gall bladder necrosis; *Candida glabrata*	E/P	N/R	No	2
19	58/M	HTN, cancer	N/R	Heparin	No	8
20	60/M	Obesity, SAD, hepatic cirrhosis, thrombocytopenia; *Candida albicans*	2	Aspirin	No	4
21	71/M	HTN, DM2, IHD, obesity, cancer, CRD	Acutely	Aspirin	No	6

Historical case data in order of 2019 autopsy case accession selected for age and comorbidities

Ethnicity: African American = 17; Caucasian = 3; Hispanic = 1

Abbreviations: HTN, systemic hypertension; DM2, diabetes mellitus type 2; DLE, discoid lupus erythematosus; DM, diabetes mellitus untyped; COPD, chronic obstructive pulmonary disease; IHD, ischemic heart disease; N/R, none recorded; NCH, not confirmed histopathologically; CRD, chronic renal disease; PE, pulmonary embolism; E/P, episodic/procedural; BD, bipolar disorder; tPA, tissue plasminogen activator; FCD, focal cortical dysplasia; HCV, hepatitis C virus infection; SAD, substance abuse disorder; CAH, chronic active hepatitis; DVT, deep vein thrombosis

By simple inspection (and by χ^2^ analysis), the two control groups, when compared to each other, had no significant difference in the rate of hypertension, acute neutrophilic vasculitis, or the combination of both hypertension and acute neutrophilic vasculitis. No transmural migration of inflammatory cells, acute perivasculitis, or paravascular hemorrhages were noted in any control brains.

For our COVID-19 cohort cases, there was no significant difference in the number of cases with hypertension (69.4%) compared to historical controls (χ^2^ = 0.004, *p* > 0.9) or to contemporary controls (χ^2^ = 0.008, *p* > 0.7) and no difference after combining both control groups for comparison (χ^2^ = 0.05, *p* > 0.8). The COVID-19 cohort also had no significant difference in the rate of diabetes mellitus from historical (χ^2^ = 1.7, *p* > 0.1) or contemporary control groups (χ^2^ = 0.007, *p* > 0.9) or in the rate of obesity for historical (χ^2^ = 0.41, *p* > 0.5) or contemporary controls (χ^2^ = 0.2, *p* > 0.6). Age range, race, and comorbidities were similar in the COVID-19 cohort and in both control groups.

Historical (9/130 = 6.9% of slides with focal or multifocal acute neutrophilic vasculitis as a proxy for disease burden) and contemporary controls (14/162 = 9% of slides) combined had significantly fewer slides with acute neutrophilic vasculitis than cohort cases, with an overall 37.8% disease burden in cohort cases (χ^2^ = 59.1, *p* < 0.00001). COVID-19 cohort cases compared to the combined historical and contemporary controls were queried for the presence of both acute neutrophilic vasculitis (disease burden) and hypertension together. Among the combined control cases, 15/48 (31.3%) had both hypertension and acute neutrophilic vasculitis compared with 25/36 (69.4%) in our COVID-19 cohort, showing a significantly higher rate in the cohort cases (χ^2^ = 4.2, *p* < 0.05).

Anti-coagulation medications were used for all COVID-19 cohort cases surviving over one day in the hospital. These included warfarin (1 case), heparin (18), apixaban (4), enoxaparin (22), clopidogrel (5), and aspirin (13). Five of these drugs were employed in one case. Tables 3 and 4 list anti-coagulation medications for control cases. Multiple contingency table comparisons of COVID-19 cohort cases with control case groups obtained both significant and non-significant results in comparisons with cases receiving warfarin, heparin, and/or apixaban including comparisons with a history of hypertension, hepatitis C virus (HCV) infection, and small brain parenchymal hemorrhages. Separate comparisons of all anti-coagulation drugs used for each case had similar mixed findings.

### Immunostaining of activated complement components

Immunostaining for complement components C3d, C4d, and C5b-9 was performed on 15 cerebral and/or brainstem sections selected for acute neutrophilic inflammatory microcirculatory findings from six COVID-19 cases. Five cases were female. COVID-19 patient comorbidities in this group included hypertension (83.3%), obesity (66.7%), ischemic heart disease (66.6%), chronic pulmonary disease (33.3%), diabetes mellitus (33.3%), psychiatric disorder (33.3%), and cancer (16.7%).

Historical control cases included 16 sections (four cerebral, four brainstem) from eight cases with death prior to December, 2019. Control case comorbidities included hypertension (75%), obesity (62.5%), diabetes mellitus (50%), ischemic heart disease (25%), psychiatric disorder (12.5%), cancer (12.5%), and alcohol abuse (12.5%). Four cases were female. The age range was 53 to 86 years.

**Fig. 5:**
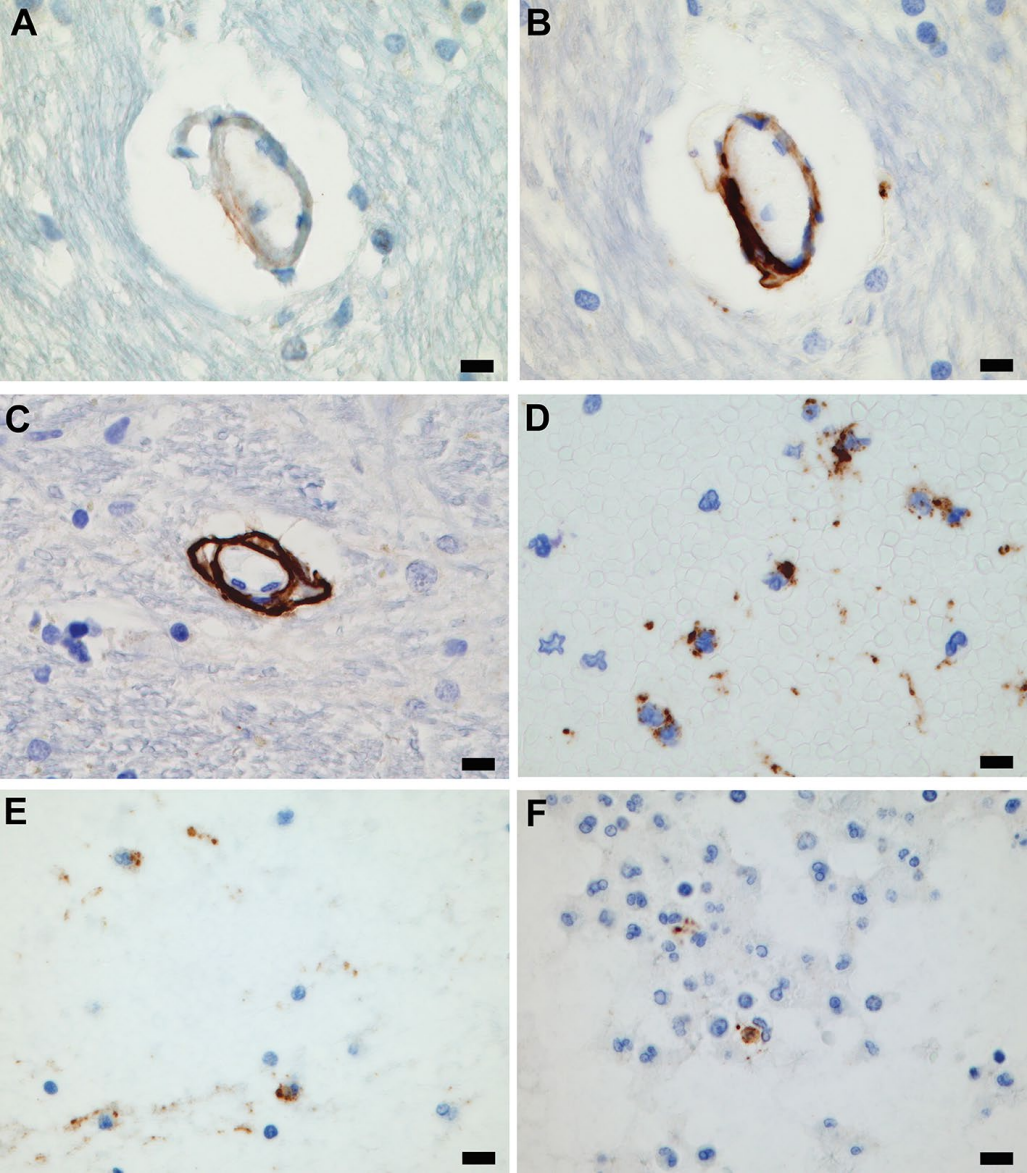
Complement component activation. (**A**) Dilated microcirculatory wall in rostral basis pontis is focally positive with immunostaining for C3d (Case 25). (**B**) Microcirculatory wall shown in (**A**) is seen here heavily positive for C4d. C5b-9 immunostaining was negative (Case 25). (**C**) Rostral basis pontis microcirculatory channel with immunostaining positive for C4d, with separation of mural layers (Case 25). (**D**) C4b positive immunostain associated with PMNs, in perivascular hemorrhage in periaqueductal central gray matter of midbrain, indicates acute perivasculitis with complement component activation (Case 4). (**E**) C5b-9 positive stain associated with PMNs in perivascular hemorrhage shown in (**D**) indicates membrane attack complex formation in acute perivasculitis (Case 4). (**F**) C5b-9 immunostaining in acute perivasculitis in rostral pontine tegmentum (Case 25). Scale bars: 10 μm in (**A**–**F**)

Formalin-fixed, paraffin-embedded sections immunostained for complement components were evaluated to determine the number of microcirculatory channel walls that were positive either focally or diffusely for any one of the three separate complement component stains from each brain tissue block. Complement component staining subtotals were combined for each tissue section as an indication of complement activation *in-situ* in that section (proxy for disease burden). Staining was zero to 40 microcirculatory channel walls in COVID-19 brain tissue sections (Fig. 5A–C). Complement component immunostaining was mostly zero in controls, with the oldest control patient (with hypertension and cancer) having 14 positive microcirculatory vessels in one section. C4d was more commonly heavily positive than C3d or C5b-9. Some microvascular walls, particularly with C4d complement component activation, appeared to have staining in separated cellular layers (Fig. 5C). The combined totals comparing all positive microcirculatory walls in COVID-19 cases with the positive channels in controls were subjected to the Mann-Whitney *U* Test which revealed a significant difference (z-ratio, 3.4; *p* < 0.01), while χ^2^ = 42.4 (*p* < 0.001).

Karyorrhectic PMNs in the COVID-19 cohort sections used for immunostaining were not positively-stained in any microcirculatory channels, where flowing blood was likely to have exchanged luminal cells prior to death. However, two of the cases had acute perivasculitis in which C4d and C5b-9 were present in association with PMNs in affected perivascular spaces (Fig. 5D–F).

### Timing of deaths in COVID-19 cohort

For the cohort, there were seven deaths between 5 and 9 AM (19.4% of cases, a period accounting for 16.6% of the day) and 12 deaths between 4 and 10 AM (33.3% of cases; 25% of the day). There was no significant difference when comparing the percentage of cases dying in these time periods (χ^2^ = 0.15; *p* > 0.6), and there was no difference when comparing all of the early AM deaths with the 17 cases not dying in the early morning hours (χ^2^ = 0.16; *p* > 0.2).

### Mechanical ventilation and pulmonary pathology findings

At autopsy, diffuse alveolar damage (DAD) was identified in 22/36 cohort cases (61.1%); hyaline membranes without an acute respiratory distress syndrome (ARDS) diagnosis, in 3/36 cases (8.3%); and ARDS, in 2/36 cases (5.6%). Brief survival (up to 6 h) following hospital admission occurred in Cases 1–6 with no history of an acute illness except for recent collapse (Table 1). All six cases were on mechanical ventilation upon hospital arrival. Pulmonary pathology showed early to somewhat advanced lung damage in these cases. Case 1, surviving for 30 min, showed patchy DAD, while the other five cases, surviving up to 6 h, showed, in case order (*i*.*e*., increasing order of time of survival), severe ARDS, early DAD, congestion and focal hemorrhage, advanced DAD, and DAD with the proliferative stage of squamous metaplasia. These pulmonary findings from the rapid deaths upon hospitalization essentially reflected the pulmonary findings in most of the other cases. Most cases surviving over one day had various stages of DAD, one (Case 18) had ARDS, and several with various times of survival had various stages of pneumonia including, for example, an acute infectious process in Case 36. Case 8 had pulmonary emboli; Case 10, marked pulmonary edema, congestion, and premortem thrombi; and Case 12, metastatic adenocarcinoma. All of these findings were dispersed through the case list with little correlation of survival time with the type or degree of pulmonary pathology. However, cases without mechanical ventilation during their hospital course tended to have less significant pulmonary disease at autopsy than cases with intubation. In Cases 7–36, intubation in the final 24 h of life (20/30 cases = 67%) reflected no more than a tendency for more severe pulmonary pathology compared to those not intubated terminally.

### Clinical and autopsy microbiology findings

In our COVID-19 cohort, the combined clinical and autopsy results for non-SARS-CoV-2 microbial agents revealed positive findings in 32/36 cases (88.9%) including all body sites. Positive bacterial cultures were present in 20/36 (55.6%) cases; fungi, in 18/36 (50%) cases; and viruses, by various detection methods, in 12/36 (33.3%) cases. The most common microbial isolate was *Enterococcus faecalis* (13/36 = 36.1% of cases), followed by *Staphylococcus aureus* (total, 12/36 = 33.3% of cases, including methicillin-resistant *Staphylococcus aureus* [13.9% of cases]), *Candida albicans* (33.3%), *Pseudomonas aeruginosa* (25%), *Klebsiella pneumoniae* (19.4%), *Streptococcus* spp. (19.4%), *Escherichia coli* (16.7%), *Candida glabrata* (16.7%), and viruses (16.7%). Coagulase negative *Staphylococcus* (25% of cases) was most likely a contaminant [27].

For the two most common non-SARS-CoV-2 species infecting the cohort, survival with a positive *E*. *faecalis* or *S*. *aureus* culture was slightly longer than the cohort mean of about 20 days. Specific compartmentalized microbiota other than those identified by routine clinical testing in the nasopharynx, blood, urine, and lung were not studied.

Case 3 had tuberculosis (Table 1), not noted in the totals above. Also not specifically noted above, but listed in Table 1, were influenza A in Case 6 and chronic HCV infections in Cases 3, 10, 20, 27, and 29 (5/36 = 13.9%). Hepatitis B virus (HBV) infections were found by history or laboratory detection in Cases 27, 28 (Table 1), 30, and 32 (4/36 = 11.1%). There was detection in cerebrospinal fluid taken clinically of varicella zoster virus in Case 1 and of cytomegalovirus and human herpesvirus 6 in Case 35.

## Discussion

The 36 autopsy cases of COVID-19 include adults from close to middle age to elderly, there is almost equal gender representation, and presentation is often with hypoxia and frequently with the diagnosis of 2019 novel coronavirus-infected pneumonia. Clinical course varies considerably in length and complexity. Most patients are hypertensive with adventitial sclerosis as evidence of microcirculatory mural injury, half have diabetes mellitus, half are obese, many have chronic heart and/or pulmonary disease, and a few have a history of cancer or other comorbidities. These are the findings in most COVID-19 patients in this age range [67, 68, 134]. Some COVID-19 comorbidities with a relatively low worldwide frequency that are included in our study groups, such as dementia, alcohol abuse, and nonalcoholic substance abuse [135], as well as HIV infection [136], are too low in case frequency for valid statistical comparisons. African Americans account for over half of the cohort cases, but given the small sample size no effect of race is inferred.

The most common clinical features among all COVID-19 cohort cases and in both of the control groups are hypertension, diabetes mellitus, and obesity, with all three of these findings occurring at the same rate in all three case groups. However, the COVID-19 cohort has a significantly higher proportion of cases with acute neutrophilic vasculitis than controls, a higher disease burden of the vasculitis in brain tissue than controls, and a significantly higher rate of hypertension plus acute neutrophilic vasculitis than the control groups. These findings suggests at least indirectly that there may be a relationship between hypertension and the histopathological finding of acute neutrophilic vasculitis with leukocytoclasia in the brain in the context of a SARS-CoV-2 infection.

### Brain microcirculatory system and microvasculopathy

Normal CNS blood vessels 40–400 μm in diameter generally are referred to as microvessels, and when including capillaries the term microcirculation is used [137]. In normal physiological and in pathological conditions, capillaries and microvessels readily dilate and the microcirculation may require further enhancement of its normal physiological remodelling. This can include the formation of IA [138, 139]. The CNS microcirculation is below direct detection by magnetic resonance imaging (MRI), although through specific imaging methods the occurrence of brain microvasculopathy, including evidence of vessel-associated hemorrhage, has been suggested in COVID-19 patients [140].

### Acute microcirculatory vasculitis in COVID-19 cohort and control cases

Acute neutrophilic microcirculatory vasculitis with leukocytoclasia is present in all 36 of the COVID-19 cohort cases. This inflammatory process also involves transmural PMN migration in some microcirculatory channels and arterioles as well as acute perivasculitis. The vasculitis is found in two of the three major brain regions (cerebrum, cerebellum, and/or brainstem) in half of the cases, including in the brainstem in all but one case. Acute neutrophilic vasculitis otherwise involves few organs in COVID-19, with most positive cases being in the skin [92, 93, 95, 96].

Acute neutrophilic vasculitis is a descriptive term that refers to leukocytoclastic vasculitis, urticarial vasculitis, and type 3 hypersensitivity vasculitis. Leukocytoclastic vasculitis is the “starting point” for many disparate types of vasculitis in a recent classification scheme [125]. The three stages of this immune-complex, small-vessel vasculitis include an initial intravascular karyorrhexis of PMN nuclei, with particles of residual chromatin material (‘nuclear dust’) briefly accumulating, accompanied by PMN transmigration and mural damage. The mural damage can include necrosis, especially in urticarial vasculitis [141, 142]. In autoimmune vasculitis, PMN activation early in this stage is the main vascular damage effector along with hypercytokinemia [122, 143]. Immunohistochemistry demonstrates mural deposition of activated complement components and often immunoglobulin in acute neutrophilic vasculitis [121, 122]. This stage continues with perivascular acute inflammation and hemorrhage, accompanied by small parenchymal hemorrhages (paravascular microhemorrhages) that can increase in size as petechial hemorrhages [93, 118–126]. This first stage includes disappearance of the apoptotic PMNs within a few days while tissue hemorrhage is still evident clinically in skin cases [122]. This is also the interval during which mural complement deposition disappears [124]. The second stage continues as a more intense necrosis of the luminal lining with dead cells, coagulated collagen, and serum proteins accumulating as a bright red ring around the vascular lumen (fibrinoid necrosis). Healing (the third stage) is by endovascular fibrosis [118, 122, 126]. Only the first stage is seen in the COVID-19 cohort and in both control groups. In the controls, only intraluminal acute neutrophilic vasculitis is found, without acute perivascular inflammation or hemorrhage and without associated parenchymal hemorrhage. The mural complement deposition in historical controls may have one or more of many causes, the most likely being hypertension.

In our COVID-19 cohort, the first stage of acute neutrophilic vasculitis has an apparent decline of acute inflammatory cells as paravascular microhemorrhage and petechial hemorrhage develop in the brain parenchyma. This is illustrated by a weak, negative correlation of these two findings (acute neutrophilic vasculitis compared with the burden of small parenchymal brain hemorrhages). This correlation, although not at a level of statistical significance, is consistent with histopathological findings expected in this type of vasculitis. That is, both acute inflammation and hemorrhage coexist in the tissue as the inflammation declines while the small, damaged blood vessels continue to extravasate blood.

*Streptococcus* spp. [121, 125], HBV, HCV [119, 121, 142, 144], and possibly *Enterococcus faecalis* [145], altogether involving 27/36 (75%) of our COVID-19 cohort, can be associated with the development of acute neutrophilic vasculitis. Generally, these are coinfections, although in other COVID-19 patients, as in our cohort, bacteremia has been considered for its likely influence on the course of the disease [13, 39, 40, 146]. This includes the influence that bacteremia can have on the development of GI tract dysbiosis [62]. For some of these microbes, an association has been made with the generation of antibodies that might lead to an immune-related vasculitis [54, 147, 148]. Other microbial associations that have been incriminated in the induction of acute neutrophilic vasculitis in the skin include *Ureaplasma urealyticum*, *Aerococcus viridans*, *Burkholderia cepacia*, *Listeria monocytogenes* [124], and dengue virus [149].

Connective tissue diseases, ANCA-associated vasculitides, cryoglobulinemic vasculitis, IgA vasculitis (Henoch-Schönlein purpura), and C1q hypocomplementemic urticarial vasculitis are other conditions that might express the histopathology of acute neutrophilic vasculitis [150]. Some of these conditions can be complicated by onset of a SARS-CoV-2 infection [151].

ANCA-associated vasculitis has presented clinical problems when already present in patients with new onset of a SARS-CoV-2 infection, while new-onset ANCA-associated vasculitis also occurs in some COVID-19 patients [152]. ANCA-associated vasculitis developing in COVID-19 patients has an association with female gender and with severe disease [151]. This type of vasculitis is associated with pauci-immune glomerulonephritis in a few patients [153, 154], one of whom also has leukocytoclastic vasculitis in a skin biopsy [153]. Leukocytoclasia characterizes the microscopic polyangiitis form of ANCA-associated vasculitis although without mural complement component deposition. The other forms of ANCA-associated vasculitis, which may have karyorrhectic PMNs, are characterized by granulomatous inflammation [121, 122].

Various medications are implicated in the development of acute neutrophilic vasculitis [121], including tocilizumab [155, 156], which was administered in four of our COVID-19 cohort cases. Acute neutrophilic vasculitis in the skin [94, 157, 158] or ANCA-associated glomerulonephritis has followed a COVID-19 vaccine [159, 160], with one of the renal biopsies showing karyorrhectic acute perivasculitis [159].

Acute neutrophilic vasculitis has a very small chance of occurring one day to about 18 months following the administration of some anti-coagulation medications, including warfarin, heparin, and apixaban [132, 133]. These and other anti-coagulants were frequently used in our COVID-19 cohort and in controls. The large proportion of hypertension in the study cases, as well as viral and bacterial associations with acute neutrophilic vasculitis, constitute confounding variables that prevent a reliable statistical association of this class of medications with vasculitis in our study.

Urticarial vasculitis does not often involve the brain [121]. A non-COVID-19 clinical report suggests indirectly that urticarial vasculitis might be present in the cerebrum in an isolated case [161]. Since urticarial vasculitis tends to cause small-vessel mural destruction, it cannot be ruled out that some of the dehiscent microcirculatory walls found in our COVID-19 cohort may at least in part represent urticarial vasculitis or theoretically, under the proper circumstances, another vasculitis such as Henoch-Schönlein purpura into which it may have evolved from its early leukocytoclastic origin [125, 141, 142].

Dehiscent microcirculatory channels may also form in a different manner in this cohort such as from hypercytokinemia, RAS dysfunction, microcirculatory remodelling, following thromboembolism [162], possibly following the effects of viral capsid proteins and/or direct infection or host response/bystander effects to viral lesions, and perhaps in a C5b-9–mediated thrombotic vascular injury syndrome (atypical hemolytic uremic syndrome or antiphospholipid antibody syndrome) [111]. An additional possible cause of necrotizing vasculitis with karyorrhectic PMNs is an *E*. *faecalis* infection [145].

The pathogenic origin of acute neutrophilic vasculitis in COVID-19 is unknown. Principal conditions speculated to underlie microcirculatory injury during the first wave of COVID-19 were hypoxia [99, 134, 163, 164], hypercytokinemia, and renin-angiotensin system (RAS) dysfunction [165]. Autoimmunity associated with hypercytokinemia or expressed as type 3 hypersensitivity vasculitis in the CNS was postulated as a potential cause of further vascular damage in COVID-19 beyond damage associated with hypoxia and host factors [68, 74, 89, 134]. Other factors that may influence the development of an autoimmune or immune-complex small-vessel vasculitis in COVID-19 patients might include environmental conditions and comorbidities such as hypertension, rheumatic conditions, cancer, and non-SARS-CoV-2 infections as discussed, as well as drugs [121, 122] and perhaps, in addition, the SARS-CoV-2 infection itself [54]. Among other difficulties in sorting out these confounding variables are the poorly-understood factors that can lead to anomalous findings in immune responses in COVID-19, particularly in immune-complex formation [115].

Immune-complex formation by the end of the second week of a SARS-CoV-2 infection, when symptoms may begin, would align with the presumed origin of acute neutrophilic vasculitis in the shortest-surviving COVID-19 cohort cases. These cases, with less than one day of hospital survival, may have been infected ten days to two weeks prior to hospitalization, although a longer, asymptomatic infection cannot be ruled out. Perhaps these early deaths with vasculitis can be at least theoretically matched to the later deaths in the cohort by a changeable window of opportunity in immune-system pressure (leading to immune-complex formation). One mechanism for such a window might arise from the periodic immunoglobulin class switching observed over the course of the first few months of COVID-19, where such switching has been suggested to affect immunity [128]. Alternatively, acute neutrophilic vasculitis in these early deaths, as in the remainder of the cohort, may either have originated through another avenue such as a comorbidity or coinfection or by a combination of these underlying factors.

### Central hypoventilation syndrome

Note is taken of the possibility of a central hypoventilation syndrome which has been suggested to occur in COVID-19 involving the brainstem’s central cardiopulmonary pacing network [166]. In this syndrome, there is failure of the switch from automatic to voluntary breathing around daybreak, and thus respiratory effort ceases as normal pacing fails from a variety of causes [167]. Failure of the switch from automatic to voluntary breathing remains a question that is not reliably supported in our small case cohort.

### Study limitations

Limitations in this study mainly involve the relatively small size of the COVID-19 autopsy cohort, with similarly small control groups. Since group comparisons are statistical, most key group similarities and differences are clear within the study. The factors in which statistical comparisons are weak or uncertain include important subjects that might help to enlighten the origin or final development of the acute neutrophilic vasculitis (viz., is it type 3 hypersensitivity vasculitis or is it developing into urticarial vasculitis?). In addition, the origin of the acute vasculitis could be related to one or more factors in the COVID-19 autopsy cases, such as a hepatitis virus or bacterial infection in conjunction with the SAR-CoV-2 infection or as a separate cause of the vasculitis. Comparison with control cases is also hampered by the relatively short survival of control cases. The number of vaccinated cases in the second year of the study is uncertain since some were admitted with very little history. Tocilizumab, COVID-19 vaccination, and anti-coagulation medications, although implicated in acute neutrophilic vasculitis, are too rarely reported in the literature for medications to be assessed adequately here, and as with other potential causes they remain confounding variables. A similar problem arises from possible influences on vasculitis by dysbiosis in various compartmentalized microbiota environments or from uncharted parameters of innate immunity markers, serum complement components, and white cell immunoglobulin receptors. Since this is not a prospective study, matching available case groups for comorbidities and treatment can only be approximated.

## Conclusion

The major observation in 36 COVID-19 autopsy brains is the finding of the first stage of acute neutrophilic vasculitis proximate to death in each case. There is a significantly lower burden of this type of vasculitis in control cases. Clinical and histopathological brain findings in the COVID-19 cases, including non-SARS-CoV-2 microbial data and general autopsy findings, provide no certain single clinicopathological pattern or tendency that might suggest a mechanism for the high level of acute neutrophilic vasculitis in COVID-19. The associations that fit this finding the best are the SARS-CoV-2 infection itself, its treatment, its sequelae, and speculative immune-system changes such as periodic alterations in neutralizing antibodies. Comorbidities (particularly hypertension) and hospitalization, particularly with mechanical ventilation, might be important factors in the origin of vasculitis or in its progression. Many of these factors are known to influence the production of an immune-complex–related microcirculatory vasculitis.

## Data Availability

The datasets created and analyzed during the current study, other than protected health information, are available from the corresponding author on reasonable request.

## Abbreviations

CNS: Central nervous system
HIV: Human immunodeficiency virus
HBV: Hepatitis B virus
HCV: Hepatitis C virus
IA: Intussusceptive arborization
MRI: Magnetic resonance imaging
PMNs: Polymorphonuclear neutrophils
RAS: Renin-angiotensin system
